# A case of avian influenza A(H5N1) in England, January 2022

**DOI:** 10.2807/1560-7917.ES.2022.27.5.2200061

**Published:** 2022-02-03

**Authors:** Isabel Oliver, Jonathan Roberts, Colin S Brown, Alexander MP Byrne, Dominic Mellon, Rowena DE Hansen, Ashley C Banyard, Joe James, Matthew Donati, Robert Porter, Joanna Ellis, Jade Cogdale, Angie Lackenby, Meera Chand, Gavin Dabrera, Ian H Brown, Maria Zambon

**Affiliations:** 1 United Kingdom Health Security Agency (UKHSA), London, United Kingdom; 2 United Kingdom Health Security Agency (UKHSA), Bristol, United Kingdom; 3 Animal and Plant Health Agency (APHA), Weybridge, United Kingdom; 4 Royal Devon and Exeter NHS Foundation Trust, Exeter, United Kingdom

**Keywords:** avian influenza, influenza A(H5N1), whole genome sequencing

## Abstract

On 5 January 2022, high pathogenicity avian influenza A(H5N1) was confirmed in an individual who kept a large flock of ducks at their home in England. The individual remained asymptomatic. H5N1 was confirmed in 19/20 sampled live birds on 22 December 2021. Comprehensive contact tracing (n = 11) revealed no additional primary cases or secondary transmissions. Active surveillance of exposed individuals is essential for case identification. Asymptomatic swabbing helped refine public health risk assessment and facilitated case management given changes in avian influenza epidemiology.

We present a case report of the first confirmed human case of avian influenza A(H5N1) in England in January 2022 following identification of the strain in a duck flock kept at their residence. We describe the clinical epidemiological and virological aspects, and discuss the importance of surveillance through an asymptomatic testing programme.

## Case detection and description

An outbreak of high pathogenicity avian influenza (HPAI) H5N1 was confirmed by the United Kingdom (UK) chief veterinary officer in a flock of ca 125 Muscovy ducks in a domestic setting in South West England on 22 December 2021. After the death of one bird on 18 December, followed by additional deaths and clinical signs of illness in the flock on 20 December, 20 Muscovy ducks were sampled on 21 December and samples submitted to the National Reference Laboratory at the Animal and Plant Health Agency (APHA), Weybridge, UK with 19 live birds testing positive for H5N1 HPAI virus.

On confirmation of the avian outbreak, the owner, in their early 80s and who had no signs of infection, was prescribed a prophylactic dose of oseltamivir (75 mg once per day) on 24 December since they had been exposed to infected birds and their secretions without personal protective equipment (PPE). On the same day, a routine nasal swab was collected from the owner as part of the UK Health Security Agency’s (UKHSA) avian influenza surveillance programme, which includes testing of asymptomatic people exposed to the virus. The sample tested positive for influenza A by PCR in two different UKHSA laboratories. Human seasonal influenza viruses, H1 and H3, were not detected by subtyping PCR assays and the circumstances in which the individual resided – close contact with avian influenza H5N1-infected birds, heavily contaminated environment and lack of plausible human contacts for seasonal influenza – led to the individual being managed as a presumptive case of avian influenza A virus. As such, the case was requested to isolate at home and received an extended treatment course of oseltamivir (75 mg twice per day) for 10 days until two consecutive PCR tests were negative at the reference laboratory; the individual remained asymptomatic throughout. Of note, the affected individual had been vaccinated on 23 November 2021 with the Fluad Tetra vaccine (Seqirus, Maidenhead, UK), containing inactivated influenza virus haemagglutinin and neuraminidase surface antigens.

## Virological investigations

The case was re-sampled with nose and throat swabs on 26 and 31 December 2021. Some of the initial PCR assays were positive for influenza A, although the cycle threshold (Ct) values were high (> 30 cycles). The UKHSA respiratory viruses unit re-extracted residual original material from 24, 26 and 31 December in order to concentrate the nucleic acid material several fold compared with standard processes; both H5 subtyping and influenza A PCR testing was repeated. Independent samples from each date confirmed influenza A and subtyping of H5, all with Ct values in the mid-30s, i.e. close to the limit of detection (40 cycles). Whole genome sequencing (WGS) of the virus strain (A/England/215201407/2021, EPI_ISL_8799552, deposited in GISAID) for influenza A was undertaken directly from human clinical respiratory material from the sample on 26 December, as previously described [1] using the Illumina platform (Illumina, San Diego, California, United States).

**Figure 1:**
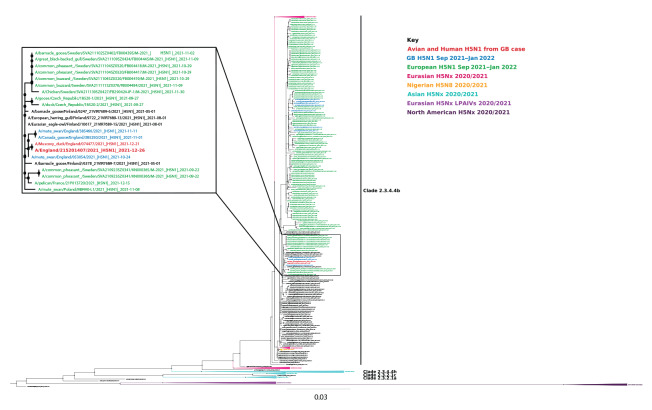
Phylogenetic tree of the identified human case with high pathogenicity avian influenza H5N1 and contemporary European sequences, England, January 2022

The H5N1 HPAI virus from the kept ducks was isolated in embryonated fowls’ eggs (A/muscovy duck/England/074477/2021) and WGS was performed (EPI_ISL_8809153). Its genomic sequence shared high homology (98.5–100% across all gene segments) and close phylogenetic relationships (7–11) with those of other H5N1 viruses detected during the ongoing UK and European epizootic in wild birds and poultry (Figure 1). 

H5 HA sequences collected between 1 January 2020 and 18 January 2022 were obtained from the EpiFlu database of the Global Initiative on Sharing Avian Influenza Data (GISAID) (Supplementary Table S1) and aligned to assess the evolutionary relationships. European H5N1 HA sequences from September 2021 to January 2022 are coloured in green, while United Kingdom H5N1 HA sequences from the same period are coloured in blue. The full coding sequences of the HA gene of the human virus (A/England/215201407/2021) and the avian virus (A/Muscovy duck/England/74477/2021) are coloured in red. Sequences were aligned using MAFFT v7.487 [11], trimming of the gene sequences to the open reading frame, removal of the secretion signal, and removal of incomplete sequences was performed using Aliview v.2019 [12] and tree generated using IQ-Tree version 2.1.4 [13] with ModelFinder [14] with 1,000 ultrafast bootstraps [15].

Sequences of all gene segments from the human virus strain (A/England/215201407/2021) were compared with those of the duck virus (A/muscovy duck/England/074477/2021). Analysis confirmed that the H5N1 in human respiratory material was identical in all segments – at the consensus level – to the avian sequence, apart from four nucleotide (nt) mutations: three synonymous mutations, two in the polymerase basic protein (PB) 2 gene at nt positions 75 and 220, and one in PB1 gene at nt position 1,481. One coding change was noted in polymerase acidic protein (PA) gene arising from a further non-synonymous mutation at nt position 485, resulting in a conservative change from asparagine (N) in the duck sequence to threonine (T) at amino acid position 162 in the human PA sequence. In all other UK H5N1 avian genomes from 2021, there is a T at position 162.

Examination of human H5N1 or H5Nx sequences in GISAID (n = 515) indicates that at PA position 162, T occurs in 97.2% while N has not been reported. In more than 9,000 avian H5N1 sequences on GISAID, N is detected at very low frequency (< 1% of sequences), suggesting that this mutation is not associated with H5N1 avian influenza viruses obtained from infected humans. Virus isolation was not possible from the H5N1-containing human materials because the original swab samples were taken into medium containing guanidinium, which inactivates live virus but preserves RNA.

Analyses of the full genome sequence of virus isolated from the ducks (A/muscovy duck/England/074477/2021) was undertaken using an international H5N1 genetic changes inventory [2] and those described by Suttie et al. 2019 [3], to identify mutations that determine viral genetic markers associated with increased virulence, potential adaptation to mammalian species or altered susceptibility to existing antiviral drugs (neuraminidase and ion channel inhibitors). The virus had the same profile as other UK H5N1 avian viruses (Figure 1) with no strong correlates for specific increased affinity for humans and contained no mutations conferring antiviral resistance to oseltamivir or amantadine.

**Figure 2:**
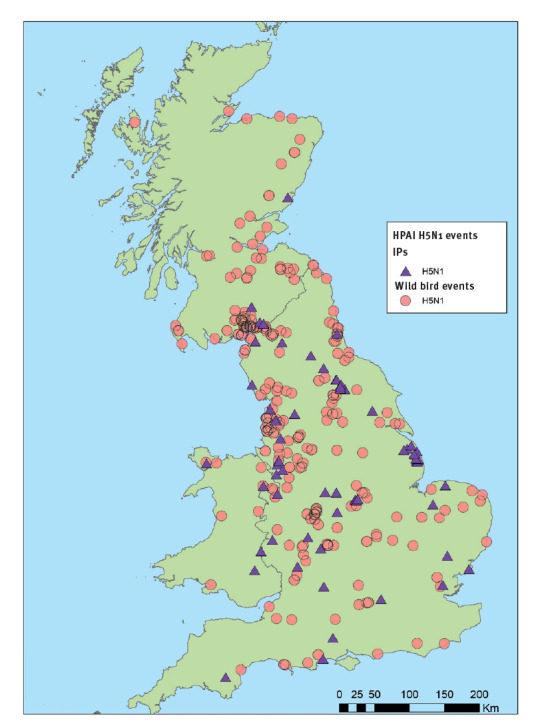
Map of high pathogenicity avian influenza H5N1-infected premises and wild bird events, Great Britain, October 2021–January 2022

HPAI: high pathogenicity avian influenza; IP: infected premises.

The figure shows Great Britain locations of high pathogenicity avian influenza A(H5N1) virus detections in either poultry at premises where birds are kept or in individual or groups of free living wild birds found dead The map was sourced from the International Disease Monitoring of the Animal and Plant Health Agency.

## Public health investigations and control measures

The public health response was coordinated through an incident management team. Of 11 contacts identified (Table), only one had close contact with the human case without the use of PPE. That contact received post-exposure antiviral prophylaxis (oseltamivir; 75 mg once per day for 10 days), tested negative following asymptomatic swabbing, and completed active monitoring for 10 days without developing symptoms.

**Table:** Management of contacts exposed to the human case with high pathogenicity avian influenza A(H5N1), England, January 2022 (n = 11)

Contact exposure	Exposed (n)	Symptomatic (n)	Active monitoring completed^a^ (n)	Prescribed antiviral drugs^b^ (n)	Asymptomatic swabbing	
					n	Test results
Without PPE	1	0	1	1	1	Neg.
With PPE	10	0	10	10	3	Neg.^c^
Total	11	0	11	11	4	NA

Neg.: negative; PPE: personal protective equipment.

^a^ The active monitoring period was 10 days after exposure and consisted of daily checks for the development of any influenza-like symptoms using an SMS text monitoring system managed by United Kingdom Health Security Agency.

^b^ Antiviral drug used was oseltamivir. No monitoring of compliance was conducted.

^c^ Asymptomatic swabbing is part of the United Kingdom Health Security Agency surveillance for individuals exposed to an avian influenza-infected premises.

The remaining 10 contacts wore PPE during all interactions with the case or the infected premises. All were placed under active monitoring for 10 days and none reported any symptoms. As all were exposed to the contaminated environment, they were offered antiviral chemoprophylaxis and participation in the UKHSA’s asymptomatic testing programme. Swabs were sent out by courier and postal kit; tests rely on self-swabbing and return. Four of 11 were returned and tested negative.

All birds at the infected premises were culled by the APHA on 30 and 31 December 2021. Professional decontamination of the whole premises including the dwelling is being undertaken.

## Epidemiological context

 Wild water birds are the reservoir for avian influenza [4]. Domestic poultry are vulnerable to spill-over infection from migratory wild birds, with outbreaks frequently reported in farm settings including a contemporaneous large outbreak in Great Britain [5]. In Great Britain, the autumn/winter 2021/22 season has seen the largest ever number of HPAI H5N1 wild bird detections and poultry outbreaks since 2003, when this avian virus started to spread from Asia to Europe and Africa. Human avian influenza infections are rare. However, some viruses, such as goose/Guangdong lineage H5Nx or Asian lineage H7N9 viruses, have been associated with human disease with high case fatality rates [6].

Since 2014, over 50 laboratory-confirmed cases of human infection with influenza A(H5N6) virus have been reported from China. The number increased during 2021, with ca 25 cases reported and 10 fatalities, emphasising the continuing high case fatality rate of zoonotic transmissions associated with H5 influenza subtypes. Virological characterisation of recent human zoonotic H5N6 viruses shows that H5N6 Eurasian viruses have a haemagglutinin (HA) protein which groups into clade 2.3.4.4h, which is distinct from the HA of the circulating European viruses, which groups into clade 2.3.3.4b [7]. HPAI H5N6 and H5N8 subtype candidate vaccine viruses have been developed or recommended for pandemic preparedness including clade 2.3.4.4b CVV (A/Astrakhan/3212/2020-like) [8]. This is of increased importance given the recent detection of clade 2.3.4.4b viruses in mammals in Europe [9].

## Ethical statement

Ethical approval was not required as this work was undertaken as part of public health response to a case of an infectious disease. The case consented to information being shared as part of a case report in the scientific literature.

## Discussion

We report the first human case of avian influenza A(H5N1) in Europe. Although transmission from birds to humans is rare, there is a risk that these viruses may adapt and become able to infect and gain the ability to spread from person to person, therefore, early identification of human infection is of great public health importance.

In the context of a high prevalence of infection in birds in England, in 2021 UKHSA strengthened its surveillance to include testing of asymptomatic, potentially exposed contacts in addition to those who report symptoms following a potential exposure event. The aim of the asymptomatic testing programme is to ensure timely and effective detection of any possible case of transmission to humans. The programme will also provide intelligence to help refine public health risk assessment and facilitate case and situation management, given these recent changes in avian influenza epidemiology. This case reported here, confirmed by PCR testing followed by WGS, was detected through this testing programme but uptake was low among the case’s contacts who used PPE. The experience from this case will be incorporated into learning for future asymptomatic swabbing programmes in Great Britain.

The wider public health risk from this particular case was assessed to be very low. The affected individual remained asymptomatic throughout the event. Virological investigations indicated low infectivity and the individual reported very limited contact with other people. Importantly, the circumstances regarding exposure to birds were unusual, with a high degree of close contact with a large number of infected birds and a virus-contaminated enclosed domestic environment which resulted in infection. The spill-over infection to the human contact did not lead to any detected genetic changes in the virus that might be associated with increased zoonotic risk. This case demonstrates the increased risk posed by this kind of close contact but does not change the overall assessment that the risk to the general public from avian influenza virus remains very low.

This case reported no symptoms at any point in time during the event. Seroprevalence studies suggest that subclinical and clinically mild human A(H5N1) virus infections are uncommon [10]. Individuals may have immunity because of prior exposure, or prompt administration of prophylactic antiviral drugs could have tempered disease progression. Focussing testing on people who develop symptoms consistent with influenza will miss some infections, with consequent risk to public health. Testing asymptomatic people accompanied with active surveillance of individuals following a high-risk exposure to the virus without any PPE protection is recommended. Such investigations increase our knowledge of the zoonotic risk posed by influenza A viruses and provide important evidence help strengthen One Health responses, particularly given the unusual infection pressure in avian populations and extensive global spread of this particular strain of H5N1.

## Supplementary Data

174000 Supplement
